# Supplementary material for: Connecting Healthcare with Income Maximisation Services: A Systematic Review on the Health, Wellbeing and Financial Impacts for Families with Young Children
Source: Int J Environ Res Public Health. 2022 May 25;19(11):6425. doi: 10.3390/ijerph19116425 (PMC9180526; doi:10.3390/ijerph19116425)
Supplement: Supplementary file 1 [file ijerph-19-06425-s001.zip › ijerph-1724904-Supplementary file S2 - Search Strategy.pdf]

Search String

In families of children ages 0-to 5 years who are experiencing financial difficulties (P,0), do health care referrals to income maximisation services (I) compared to usual care (C) have a positive impact on family finances, and/or parent/caregiver, and/or child health and wellbeing (O).

Limits:
Language: English;
Age: expectant mothers/families/parents/guardians/or carers of children ages 0-to 5 years;
Record Type: academic journals; grey literature
Date: No limits.

Data Base
**MEDLINE (Ovid)**
exp family/ OR (parent* OR guardian* OR carer* OR mother* OR father* OR grandparent* OR child* OR prenatal OR pregnan* OR famil*).mp
AND (Financial adj2 (counsel* OR education OR therap* OR service* OR advice OR advisor* OR literacy OR support OR benefit* OR difficul* OR hardship* OR stress* OR pressure* OR problem OR instabilit*) OR (Income maximi* OR income advice OR debt counsel* OR welfare benefit maximi* OR welfare advice* OR money advice worker* OR welfare right* OR local government welfare officer* OR application assistance OR reduc* allocation cost* OR welfare take-up OR debt minimi* OR income management OR reduc* application cost* OR poverty minimi*)).mp.
AND exp maternal health services/ OR Public Health Nursing/ OR Community Health Nursing/ OR social worker/ OR general practitioner/ OR Pediatrician/ OR Physicians/ OR Physicians, Family/ OR Physicians, Primary Care/ OR Physicians, Women/ OR midwifery/ OR primary health care/ OR secondary health care/ OR tertiary health care/ OR patient care/ OR<br>(doctor* OR health nurs* OR clinician* OR community health worker* OR family centred care OR GPs OR health personnel OR health provider* OR healthcare provider* OR health-care provider* OR health worker* OR healthcare worker* OR health-care worker* OR health service provider* OR health service professional* OR health professional* OR healthcare professional* OR health-care professional* OR health practitioner* OR healthcare practitioner* OR health-care practitioner* OR health care service OR integrated health care delivery OR nurs* OR maternal health service* OR midwi?e* OR nurse-midwife OR patient navigat* OR physician* OR primary health care OR primary healthcare OR pediatrician* OR paediatrician* OR pediatric* care OR paediatric* care SOR primary care provider* OR practitioner* OR secondary health care OR secondary healthcare OR shared services health care OR social worker* OR secondary care provider* OR tertiary health care OR tertiary healthcare OR tertiary care provider* OR patient navigat*).mp

EMBASE (Ovid)
family/ OR exp nuclear family/ or single-parent family/ or (parent* OR guardian* OR carer* OR mother* OR father* OR grandparent* OR child* OR prenatal OR pregnan* OR famil*).mp
AND ((Financial adj2 (counsel* OR education OR therap* or service* OR advice or advisor* OR literacy OR support OR benefit* OR difficul* OR hardship* OR stress* OR pressure* OR problem* OR instabili*)) OR (Income maximi* OR income advice OR debt counsel* OR welfare benefit maximi* OR welfare advice* OR money advice worker* OR welfare right* OR local government welfare officer* OR application assistance OR reduc* allocation cost* OR welfare take-up OR debt minimi* OR income management OR reduc* application cost* OR poverty minimis*)).mp.
AND exp maternal health services/ OR Public Health Nursing/ OR Exp Community Health Nursing/ OR social worker/ OR general practitioner/ OR Pediatrician*/ OR Physicians/ OR midwifery/ OR nurse/ OR expert nurse/ OR nurse consultant/ OR nurse researcher/ OR practical nurse/ OR registered nurse/ OR staff nurse/ OR primary health care/ or secondary care/ OR tertiary healthcare/ OR patient navigation/ OR<br><br>(doctor* OR health nurs* OR clinician* OR community health worker* OR family centred care OR GPs OR health personnel OR health provider* OR healthcare provider* OR health-care provider* OR health worker* OR healthcare worker* OR health-care worker* OR health service provider* OR health service professional* OR health professional* OR healthcare professional* OR health-care professional* OR health practitioner* OR healthcare practitioner* OR health-care practitioner* OR health care service OR integrated health care delivery OR nurs* OR maternal health service* OR midwi?e* OR nurse-midwife OR patient navigat* OR physician* OR primary health care OR primary healthcare OR pediatrician* OR paediatrician* OR pediatric* care OR paediatric* care OR primary care provider* OR practitioner* OR secondary health care OR secondary healthcare OR shared services health care OR social worker* OR secondary care provider* OR tertiary health care OR tertiary healthcare OR tertiary care provider* OR patient navigat*).mp

CINAHL (EBSCO)	
(MH Family) OR (MH "Nuclear Family+") OR parent* OR guardian* OR carer* OR mother* OR father* OR grandparent* OR child* OR prenatal OR pregnan* OR famil*	
AND (Financial N2 (counsel* OR education OR therap* OR service* OR advice OR advisor OR literacy OR support OR benefit* OR difficul* OR hardship* OR stress* OR pressure* OR problem* OR instabilit*)) OR "Income maximi*" OR "income advice" OR "debt counsel*" OR "welfare benefit maximi*" OR "welfare advice*" OR "money advice worker"* OR "welfare rights*" OR "local government welfare officer*" OR "application assistance" OR "reduc* allocation cost*" OR "welfare take-up" OR "debt minimi*" OR "income management" OR "reduc* application cost*" OR "poverty minimi*" OR (MH "Maternal-Child Welfare") OR (MH "Maternal Welfare")	
AND (MH "Primary Health Care") OR (MH "Secondary Health Care") OR (MH "Tertiary Health Care") OR (MH "Physicians, Family") OR (MH "Physicians") OR (MH "Community Health Workers") OR (MH "Nurses") OR (MH "Midwives") OR (MH "Maternal Health Services") OR (MH	

"Community Health Nursing+") OR (MH "Family Nursing") OR (MH "Maternal-Child Nursing") OR (MH "Pediatricians") OR (MH "Physicians, Family") OR (MH "Physicians, Women") OR (MH "Physicians") OR (MH "Midwifery") OR (MH "Nurse Midwifery") OR (MH "Patient Navigation") OR (MH "Health Care Delivery, Integrated") OR (MH "Family Centered Care+") OR (MH "Shared Services, Health Care") OR<br><br>(doctor* OR health nurs* OR clinician* OR "community health worker*" OR "family centred care" OR GPs OR "health personnel" OR "health provider*" OR "healthcare provider*" OR "health-care provider*" OR "health worker*" OR "healthcare worker*" OR "health-care worker*" OR "health service provider*" OR "health service professional*" OR "health professional*" OR "healthcare professional*" OR "health-care professional*" OR "health practitioner*" OR "healthcare practitioner*" OR "health-care practitioner*" OR "health care service" OR "integrated health care delivery" OR nurs* OR "maternal health service*" OR midwi?e* OR "nurse-midwife" OR "patient navigat*" OR physician* OR "primary health care" OR "primary healthcare" OR pediatrician* OR paediatrician* OR "pediatric* care" OR "paediatric* care" OR "primary care provider*" OR practitioner* OR "secondary health care" OR "secondary healthcare" OR "shared services health care" OR "social worker*" OR "secondary care provider*" OR "tertiary health care" OR "tertiary healthcare" OR "tertiary care provider*" OR "patient navigat*").mp
Psycinfo (Ovid)
Family/ or parents/ OR single parents/ parent* OR guardian* OR carer* OR mother* OR father* OR grandparent* OR child* OR prenatal OR pregnan* OR famil*).mp
AND (Financial adj2 (counsel* OR education OR therap* OR service* OR advice OR advisor* OR literacy OR support OR benefit* OR difficul* OR hardship* OR stress* OR pressure* OR instability*) OR Income maximi* OR income advice OR debt counsel* OR welfare benefit maximi* OR welfare advice* OR money advice worker* OR welfare rights* OR local government welfare officer* OR application assistance OR welfare application assistance OR reduc* allocation cost* OR welfare take-up OR debt minimi* OR income management OR reduc* application cost* OR poverty minimi*.mp.
AND Health care services/ OR Public Health service Nurses/ OR social workers/ *OR* general practitioners/ OR family physicians/ OR primary health care/ OR Pediatricians/ OR Physicians/ OR (doctor* OR health nurs* OR clinician* OR community health worker* OR family centred care OR GPs or health personnel OR health provider* OR healthcare provider* OR health-care provider* OR health worker* OR healthcare worker* OR health-care worker* OR health service provider* OR health service professional* OR health professional* OR healthcare professional* OR health-care professional* OR health practitioner* OR healthcare practitioner* OR health-care practitioner* OR health care service OR integrated health care delivery OR nurs* OR maternal health service* OR midwi?e* OR nurse-midwife OR patient navigat* OR physician* OR primary health care OR primary healthcare OR pediatrician* OR paediatrician* OR pediatric* care OR paediatric* care OR

primary care provider* OR practitioner* OR secondary health care OR secondary healthcare OR shared services health care OR social worker* OR secondary care provider* OR tertiary health care OR tertiary healthcare OR tertiary care provider* OR patient navigat*).mp
**Emcare (Ovid)**
exp nuclear family/ OR family/ OR (parent* OR guardian* OR carer* OR mother* OR father* OR grandparent* OR child* OR prenatal OR pregnan* OR famil*).mp
AND (Financial adj2 (counsel* OR education OR therapy OR service* OR advice OR advisor* OR literacy OR support OR benefit* OR difficul* OR hardship* OR stress OR pressure* OR pressure* OR instability) OR (Income maximi* OR income advice OR debt counsel* OR welfare benefit maximi* OR welfare advice* OR money advice worker* OR welfare rights* OR local government welfare officer* OR application assistance OR welfare application assistance OR reducing allocation cost* OR welfare take-up OR debt minimisation OR income management OR reducing application cost* OR poverty minimis*)).mp.
AND maternal health services/ OR Community Health Nursing/ OR social worker/ OR general practitioner/ OR Pediatrician/ OR Physicians/ OR female physician/ OR midwife/ OR primary health care/ OR secondary health care/ OR tertiary health care/ OR patient navigation/ OR (doctor* OR health nurs* OR clinician* OR community health worker* OR family centred care OR GPs or health personnel OR health provider* OR healthcare provider* OR health-care provider* OR health worker* OR healthcare worker* OR health-care worker* OR health service provider* OR health service professional* OR health professional* OR healthcare professional* OR health-care professional* OR health practitioner* OR healthcare practitioner* OR health-care practitioner* OR health care service OR integrated health care delivery OR nurs* OR maternal health service* OR midwi?e* OR nurse-midwife OR patient navigat* OR physician* OR primary health care OR primary healthcare OR pediatrician* OR paediatrician* OR pediatric* care OR paediatric* care OR primary care provider* OR practitioner* OR secondary health care OR secondary healthcare OR shared services health care OR social worker* OR secondary care provider* OR tertiary health care OR tertiary healthcare OR tertiary care provider* OR patient navigat*).mp
**Informit online**
family OR parent* OR guardian* OR carer* OR mother* OR father* OR grandparent* OR child* OR prenatal OR pregnan*<br>AND<br>"Financial counsel*" OR "financial education" OR "financial therapy" OR "financial service*" OR "financial advice" OR "financial advisor*" OR "financial literacy" OR "financial support" OR "financial benefit*" OR "financial difficul*" OR "financial hardship*" OR "financial stress*" OR "financial pressure*" OR "financial problem*" OR "financial instabilit*" OR "Income maximi*" OR "income advice" OR "debt counsel*" OR "welfare benefit maximi*" OR "welfare advice*" OR "money advice worker*" OR "welfare right*" OR "local government welfare officer*" OR

"application assistance" OR "welfare application assistance" OR "reduc* allocation cost*" OR "welfare take-up" OR "debt minimi*" OR "income management" OR "reduc* application cost*" OR "poverty minimi*" OR "Maternal-Child Welfare" OR "Maternal Welfare"
AND
doctor* OR "health nurs*" OR clinician* OR "community health worker*" OR "family centred care" OR GPs OR "health personnel" OR "health provider*" OR "healthcare provider*" OR "health-care provider*" OR "health worker*" OR "healthcare worker*" OR "health-care worker*" OR "health service provider*" OR "health service professional*" OR "health professional*" OR "healthcare professional*" OR "health-care professional*" OR "health practitioner*" OR "healthcare practitioner*" OR "health-care practitioner*" OR "health care service" OR "integrated health care delivery" OR nurs* OR "maternal health service*" OR midwi?e* OR "nurse-midwife" OR "patient navigat*" OR physician* OR "primary health care" OR "primary healthcare" OR pediatrician* OR paediatrician* OR "pediatric* care" OR "paediatric* care" OR "primary care provider*" OR practitioner* OR "secondary health care" OR "secondary healthcare" OR "shared services health care" OR "social worker*" OR "secondary care provider*" OR "tertiary health care" OR "tertiary healthcare" OR "tertiary care provider*" OR "patient navigat*"

Family & Society Studies	

family OR parent* OR guardian* OR carer* OR mother* OR father* OR grandparent* OR child* OR prenatal OR pregnan*
AND
Financial N2 counsel* OR Financial N2 education OR Financial N2 therapy OR Financial N2 service* OR Financial N2 advice OR Financial N2 advisor* OR Financial N2 literacy OR Financial N2 support OR Financial N2 benefit* OR Financial N2 difficul* OR Financial N2 hardship* OR Financial N2 stress* OR Financial N2 pressure* OR Financial N2 problem* OR Financial N2 instabilit* OR “Income maximi*” OR “income advice” OR “debt counsel*” OR “welfare benefit maximi*” OR “welfare advice*” OR “money advice worker*” OR “welfare right*” OR “local government welfare officer*” OR “application assistance” OR “welfare application assistance” OR “reducing allocation cost*” OR “welfare take-up” OR “debt minimisation” OR “income management” OR “reducing application cost*” OR “poverty minimis*” or “Maternal-Child Welfare” OR “Maternal Welfare”
AND
doctor* OR “health nurs*” OR clinician* OR “community health worker*” OR “family centred care” OR GPs OR “health personnel” OR “health provider*” OR “healthcare provider*” OR “health-care provider*” OR “health worker*” OR “healthcare worker*” OR “health-care worker*” OR “health service provider*” OR “health service professional*” OR “health professional*” OR “healthcare professional*” OR “health-care professional*” OR “health practitioner*” OR “healthcare practitioner*” OR “health-care practitioner*” OR “health care service” OR “integrated health care delivery” OR nurs* OR “maternal health service*” OR midwi?e* OR “nurse-midwife” OR “patient navigat*” OR physician* OR “primary health care” OR “primary healthcare” OR pediatrician* OR paediatrician* OR “pediatric* care” OR “paediatric* care” OR “primary care provider*” OR practitioner* OR “secondary health care” OR “secondary healthcare” OR “shared services health care” OR

“social worker*” OR “secondary care provider*” OR “tertiary health care” OR “tertiary healthcare” OR “tertiary care provider*” OR “patient navigat*”
**Cochrane**
family OR parent* OR guardian* OR carer* OR mother* OR father* OR grandparent* OR child* OR prenatal OR pregnan*
AND ‘’Financial counsel*” OR “financial education” OR “financial therapy” OR “financial service*” OR “financial advice” OR “financial advisor*” OR “financial literacy” OR “financial support” OR “financial benefit*” OR “financial difficul*” OR “financial hardship*” OR “financial stress*” OR “financial pressure*” OR “financial problem*” OR “financial instabilit*” or “Income maximi*” OR “income advice” OR “debt counsel*” OR “welfare benefit maximi*” OR “welfare advice*” OR “money advice worker*” OR “welfare right*” OR “local government welfare officer*” OR “application assistance” OR “welfare application assistance” OR “reduc* allocation cost*” OR “welfare take-up” OR “debt minimi*” OR “income management” OR “reduc* application cost*” OR “poverty minimi*” or “Maternal-Child Welfare" OR "Maternal Welfare"
AND doctor* OR “health nurs*” OR clinician* OR “community health worker*” OR “family centred care” OR GPs OR “health personnel” OR “health provider*” OR “healthcare provider*” OR “health-care provider*” OR “health worker*” OR “healthcare worker*” OR “health-care worker*” OR “health service provider*” OR “health service professional*” OR “health professional*” OR “healthcare professional*” OR “health-care professional*” OR “health practitioner*” OR “healthcare practitioner*” OR “health-care practitioner*” OR “health care service” OR “integrated health care delivery” OR nurs* OR “maternal health service*” OR midwi?e* OR “nurse-midwife” OR “patient navigat*” OR physician* OR “primary health care” OR “primary healthcare” OR pediatrician* OR paediatrician* OR “pediatric* care” OR “paediatric* care” OR “primary care provider*” OR practitioner* OR “secondary health care” OR “secondary healthcare” OR “shared services health care” OR “social worker*” OR “secondary care provider*” OR “tertiary health care” OR “tertiary healthcare” OR “tertiary care provider*” OR “patient navigat*”
**Proquest Databases**
family OR parent* OR guardian* OR carer* OR mother* OR father* OR grandparent* OR child* OR prenatal OR pregnan*<br>AND<br>“Financial counsel*” OR “financial education” OR “financial therapy” OR “financial service*” OR “financial advice” OR “financial advisor*” OR “financial literacy” OR “financial support” OR “financial benefit*” OR “financial difficul*” OR “financial hardship*” OR “financial stress*” OR “financial pressure*” OR “financial problem*” OR “financial instabilit*” OR “Income maximi*” OR “income advice” OR “debt counsel*” OR “welfare benefit maximi*” OR “welfare advice*” OR “money advice worker*” OR “welfare right*” OR “local government welfare officer*” OR

“application assistance” OR “welfare application assistance” OR “reduc* allocation cost*” OR “welfare take-up” OR “debt minimi*” OR “income management” OR “reduc* application cost*” OR “poverty minimi*” or “Maternal-Child Welfare” OR “Maternal Welfare”

AND

doctor* OR “health nurs*” OR clinician* OR “community health worker*” OR “family centred care” OR GPs OR “health personnel” OR “health provider*” OR “healthcare provider*” OR “health-care provider*” OR “health worker*” OR “healthcare worker*” OR “health-care worker*” OR “health service provider*” OR “health service professional*” OR “health professional*” OR “healthcare professional*” OR “health-care professional*” OR “health practitioner*” OR “healthcare practitioner*” OR “health-care practitioner*” OR “health care service” OR “integrated health care delivery” OR nurs* OR “maternal health service*” OR midwi?e* OR “nurse-midwife” OR “patient navigat*” OR physician* OR “primary health care” OR “primary healthcare” OR pediatrician* OR paediatrician* OR “pediatric* care” OR “paediatric* care” OR “primary care provider*” OR practitioner* OR “secondary health care” OR “secondary healthcare” OR “shared services health care” OR “social worker*” OR “secondary care provider*” OR “tertiary health care” OR “tertiary healthcare” OR “tertiary care provider*” OR “patient navigat*”
